# Nutritional Status and Its Impact on Treatment Tolerance in Non-Small-Cell Lung Cancer Patients Receiving Osimertinib

**DOI:** 10.3390/nu17050927

**Published:** 2025-03-06

**Authors:** Claudia Barca-Díez, Regina Palmeiro-Carballa, Susana Castro-Luaces, Maria Susana Fortes-González, Silvia Vazquez-Blanco, Noemi Martínez-López-De-Castro, Natividad Lago-Rivero

**Affiliations:** 1 Pharmacy Department, University Hospital Complex of Vigo, 36312 Vigo, Spain; susana.castro.luaces@sergas.es (S.C.-L.); maria.susana.fortes.gonzalez@sergas.es (M.S.F.-G.); noemi.martinez.lopezdecastro@sergas.es (N.M.-L.-D.-C.); natividad.lago.rivero@sergas.es (N.L.-R.); 2 Pharmacy Department, University Hospital Complex of Pontevedra, 36071 Pontevedra, Spain; 3 Endocrinology and Nutrition Department, University Hospital Complex of Vigo, 36312 Vigo, Spain; regina.palmeiro.carballeira@sergas.es; 4 Innovation in Clinical Pharmacy Research Group (i-FARMA-Vigo), Galicia Sur Health Research Institute (IIS Galicia Sur), SERGAS-UVIGO, 36312 Vigo, Spain; 5 Pharmacy Department, Hospital Ribera Povisa, 36211 Vigo, Spain; svazquezb@povisa.es

**Keywords:** non-small-cell lung cancer, osimertinib, malnutrition, body composition, dose-limiting toxicities, sarcopenia, dynapenia

## Abstract

**Background/Objectives**: Non-small-cell lung cancer (NSCLC) accounts for approximately 85% of all lung cancer cases and is a leading cause of morbidity and mortality worldwide. Between 35% and 65% of NSCLC patients experience nutritional problems or malnutrition, which significantly affects their prognosis and quality of life. This study aims to describe the nutritional status and body composition of NSCLC patients treated with osimertinib, an oral tyrosine kinase inhibitor, while also assessing the prevalence of sarcopenia, presarcopenia, and dynapenia. Additionally, we explore the relationship between dose-limiting toxicities (DLTs) and nutritional status, as well as the impact of nutritional status on quality of life using the EQ-5D scale. **Methods**: A cross-sectional, observational study was conducted in 25 adult patients diagnosed with NSCLC treated with osimertinib under pharmacotherapeutic follow-up in the Pharmacy Consultations of a tertiary-level hospital. Anthropometric parameters, body composition (via bioimpedance analysis), and muscle functionality (via dynamometry) were assessed. Quality of life was evaluated using the EQ-5D scale. **Results**: The results showed that 36% of patients were malnourished, 4% had sarcopenia, 8% were presarcopenic, and 20% had dynapenia. Patients with DLTs exhibited significantly lower values of fat-free mass and the fat-free mass index, suggesting a relationship between low muscle mass and increased toxicities. **Conclusions**: These findings underscore the importance of early, individualized nutritional interventions in NSCLC patients treated with osimertinib to improve nutritional status and optimize oncological outcomes. Further research with larger cohorts and longitudinal designs is necessary to confirm these findings.

## 1. Introduction

Non-small-cell lung cancer (NSCLC) represents approximately 85% of all lung cancer cases and is one of the leading causes of morbidity and mortality worldwide. At the time of diagnosis, 35% to 65% of patients with non-small-cell lung cancer (NSCLC) exhibit nutritional challenges or signs of malnutrition. These nutritional problems significantly influence the prognosis, treatment, and quality of life of patients, regardless of the disease’s stage or the tumor’s histological type [1,2].

The main nutritional problem in oncological patients, and probably the one with the greatest impact on prognosis, is sarcopenia. Sarcopenia is characterized by a loss of skeletal muscle mass (SMM) and a decrease in muscle strength. According to the European Working Group on Sarcopenia in Older People (EWGSOP), sarcopenia is a fundamental part of cancer cachexia, which directly or indirectly accounts for the death of a third of cancer patients and is a crucial component in evaluating oncological patients [3].

Regarding the different degrees of sarcopenia, the EWGSOP group proposes a conceptual classification into “presarcopenia”, where there is only a decrease in muscle mass; “sarcopenia”, where muscle mass decrease is accompanied by either the loss of muscle strength (decreased functional capacity) or reduced physical performance; and “severe sarcopenia”, where all three of the following parameters coexist: loss of muscle mass, muscle strength, and physical performance [3].

Muscle loss is present in more than 50% of newly diagnosed cancer patients and is related to the catabolic effects of cancer-induced inflammation, such as decreased protein synthesis, increased muscle proteolysis, and hypermetabolism. Furthermore, muscle loss is exacerbated by common side effects of oncological treatment, such as nausea and loss of appetite, which reduce patients’ caloric and protein intake. Low muscle mass has been recognized as a prognostic factor for morbidity and mortality in various malignancies, including lung cancer [4].

Tyrosine kinase inhibitors (TKIs) are effective agents in a wide range of tumors, including NSCLC with epidermal growth factor receptor (EGFR) mutations. The mechanism of action of TKIs is based on their ability to block the activity of tyrosine kinases, which are enzymes involved in activating various intracellular molecular pathways related to cell signaling and tumor cell growth. However, interference with these anabolic pathways can also negatively affect normal tissues, including skeletal muscle. The PI3K/AKT/mTOR pathway, essential for protein synthesis and muscle maintenance, or the AKT/mTOR pathway, which is involved in regulating skeletal muscle fiber size, may be compromised during TKI treatment, reducing the muscle’s ability to synthesize new proteins [4,5]. Furthermore, the guidelines of the European Society for Clinical Nutrition and Metabolism (ESPEN) identify weight loss as a common side effect of targeted therapies, with multikinase inhibitors being associated with skeletal muscle atrophy.

For this reason, muscle loss during TKI therapy has become a significant clinical concern, as the onset of sarcopenia in these patients could lead to dose-limiting toxicities (DLTs), defined as any toxicity that results in a dose reduction, a temporary interruption of treatment, or the permanent discontinuation of treatment. These toxicities can compromise the effectiveness and safety of the treatment [4,6]. Several studies have evaluated the relationship between low muscle mass during TKI therapy and increased toxicity, as well as worse survival outcomes. This effect has been demonstrated in patients treated with sorafenib, but it remains almost unexplored in patients undergoing treatment with other third-generation TKIs, such as osimertinib [4,5,7,8,9].

Osimertinib is a potent, irreversible oral TKI that selectively targets both sensitizing EGFR mutations (EGFRms) and the T790M resistance mutation, with approval for monotherapy as a form of adjuvant treatment after complete tumor resection in adult patients with stage IB-IIIA NSCLC whose tumors present deletions in exon 19 of EGFR or exon 21 (L858R) substitution mutations. It is also used as first-line treatment for adults with locally advanced or metastatic NSCLC with activating EGFR mutations and in patients with favorable T790M EGFR mutations following first-line treatment with EGFR-TKI [10].

Given the above and the high prevalence of nutritional problems in patients diagnosed with lung cancer, we decided to conduct a nutritional assessment of patients diagnosed with NSCLC treated with osimertinib at our center. The primary objective was to determine the nutritional status and body composition of these patients, as well as to establish the prevalence of sarcopenia and observe whether there was a relationship between the occurrence of DLTs during treatment and patients’ nutritional status. Additionally, we evaluated the quality of life of the patients using the EQ-5D scale to identify the impact of nutritional status on quality of life during osimertinib treatment.

## 2. Materials and Methods

### 2.1. Study Design and Population

An observational, descriptive, and cross-sectional study was conducted with a cohort of 25 adult patients diagnosed with NSCLC and treated with osimertinib monotherapy under pharmacotherapeutic follow-up in the outpatient clinics of the Pharmacy Department at a tertiary care hospital.

The inclusion criteria were as follows: patients aged 18 years or older, diagnosed with NSCLC, undergoing treatment with osimertinib, retrieving their medication from the outpatient Pharmacy Consultation, and having provided written informed consent to participate in the study. Patients were excluded if they had discontinued osimertinib treatment for any reason before the nutritional assessment, were receiving osimertinib for indications not approved in the official prescribing information, or were unable to undergo the nutritional assessment due to medical or logistical reasons. Withdrawal criteria included the patient’s revocation of consent to participate in the study.

Patients were recruited during routine pharmacy practice in the outpatient Pharmacy Consultations between February and May 2024. Informed consent was provided to all patients actively receiving osimertinib who met the inclusion criteria and none of the exclusion criteria.

### 2.2. Nutritional Assessment

All patients who agreed to participate in the study underwent a nutritional assessment by the hospital pharmacist during their next scheduled visit for medication collection at the outpatient Pharmacy Consultations.

#### 2.2.1. Demographic and Clinical Data

Demographic and clinical data were obtained from patients’ electronic medical records. The variables collected included age, sex, diagnosis, stage of NSCLC, start date of osimertinib treatment, initial osimertinib dose, current osimertinib dose, total duration of osimertinib treatment, and DLTs experienced during treatment.

A total of 25 patients were included in the study: 60% were female (*n* = 15), and 40% were male (*n* = 10), with a median age of 72 years (33–87). The majority of patients (96%, *n* = 24) had adenocarcinoma, while 4% (*n* = 1) had squamous cell carcinoma. In terms of disease stage, 72% (*n* = 18) were diagnosed at stage IV, and 28% (*n* = 7) were diagnosed at stage III.

All patients started osimertinib at 80 mg/day, but 52% (*n* = 13) required dose adjustments due to toxicity. The median duration of treatment was 472 days (42–1526).

The most common DLTs included diarrhea (20%), mucositis (8%), folliculitis (8%), nausea (4%), thrombocytopenia (4%), paronychia (4%), and elevated transaminases (4%).

#### 2.2.2. Anthropometric Measurements

The following measurements were taken for all study patients as follows: current weight (kg), height (m), calf circumference (CC) (cm), and body mass index (BMI) (kg/m^2^).

The reference values for BMI used were as follows: <18.5 kg/m^2^ underweight; between 18.5 kg/m^2^ and 24.9 kg/m^2^ normal weight; between 25.0 kg/m^2^ and 29.9 kg/m^2^ overweight; and ≥30.0 kg/m^2^ obesity [11].

The limits for muscle mass reduction based on calf circumference measurements were as follows: <33 cm for men and <32 cm for women [12].

The tools used for anthropometric data collection included a weighing scale with an integrated stadiometer (Asimed^®^ brand, manufactured by Sibelmed, Barcelona, Spain) and an anthropometric measuring tape.

During the consultation, the following variables were recorded through a patient interview: usual weight, weight loss during treatment, and food intake recall.

Weight loss was calculated using the following formula: %WL = [(usual weight − current weight)/usual weight] × 100. In cases where precise information was unavailable in the medical record, weight loss was based on the comparison between the usual weight provided by the patient and the current weight measured during the consultation without being able to determine the exact time period in which it occurred.

#### 2.2.3. Body Composition Analysis by Bioimpedance (BIA)

For the body composition assessment, the InBody S10^®^ body composition analyzer (manufactured by InBody Co., Ltd., Seoul, Republic of Korea), was used, which is a device that measures impedance using 6 frequencies (1, 5, 50, 250, 500, and 1000 kHz) for each of the 5 body segments: right arm, left arm, trunk, right leg, and left leg.

The measurements were performed with patients standing barefoot, with their legs apart and arms not touching the torso. The 8-point tactile electrodes were placed on the thumb and middle finger of each hand and on both ankles. The data obtained were analyzed using the LookinBody 120^®^ software (version 4.0.0.7) to estimate body composition in terms of fat mass (FM), lean body mass (LBM), and lean body mass index (LBMI).

A reduced muscle mass was considered for LBMI < 17 kg/m^2^ in men and <15 kg/m^2^ in women [12,13].

As BIA relies on stable hydration status for accurate estimations, its precision may be affected by fluid imbalances and inflammation, which are common in oncology patients. This should be taken into account when interpreting the results.

To ensure measurement reliability, patients were instructed to follow standard pre-test recommendations, including fasting for 3–4 h before the test, avoiding strenuous exercise for at least 12 h prior to the measurement, and emptying the bladder before the test. Women were advised not to undergo BIA testing during menstruation due to potential fluid retention effects.

#### 2.2.4. Muscle Functionality Analysis by Hand Grip Strength

Muscle functionality was assessed through hand grip strength using a hydraulic dynamometer (Jamar^®^, manufactured by Performance Health, Warrenville, IL, USA), providing the strength of the dominant arm (kg).

Three measurements were taken with the subject seated and without arm support, with a 60 s rest between each measurement to prevent muscle fatigue.

Dynapenia or reduced strength was considered if the value was <27 kg in men and <16 kg in women [3].

### 2.3. Diagnosis of Malnutrition and Sarcopenia

For the diagnosis of malnutrition, the following GLIM criteria (Global Leadership Initiative on Malnutrition) were applied [13]:

Phenotypic Criteria:

- Involuntary weight loss: >5% in the last 6 months or >10% in more than 6 months.
- Low BMI: <20 kg/m^2^ if <70 years or <22 kg/m^2^ if ≥70 years.
- Muscle mass evaluation: assessed by LBMI (BIA) or by CC if BIA was not performed.
- Etiological Criteria:
- Reduced food intake or absorption: ≤50% of energy requirements for more than 1 week, any reduction for more than 2 weeks, or any chronic gastrointestinal condition negatively affecting food absorption or assimilation.
- Inflammation or acute/chronic disease: the presence of inflammatory conditions or chronic diseases affecting nutritional status.

The combination of at least one phenotypic criterion and one etiological criterion enabled the diagnosis of malnutrition. The etiological criterion of inflammation, required for the diagnosis of malnutrition, was met in all study patients as they were diagnosed with NSCLC.

For sarcopenia detection and diagnosis, the EWGSOP2 algorithm proposed by the European Working Group on Sarcopenia in Older People (EWGSOP) was applied [3]. The screening was performed using the Strength, Assistance walking, Rise from a chair, Climb stairs, and Falls (SARC-F) questionnaire, and a score of 4 or higher was considered to indicate a risk of sarcopenia. Muscle strength was determined by dynamometry, and BIA or CC assessed muscle mass. Patients with low muscle strength were classified as dynapenic; presarcopenia was diagnosed in patients showing reduced muscle mass and no significant decrease in muscle strength; finally, sarcopenia was diagnosed in patients who, in addition to low hand grip strength, also exhibited a reduction in muscle mass.

### 2.4. Quality of Life Questionnaire

The quality of life of the patients was assessed using the EQ-5D scale, a standardized tool that measures five dimensions of health: mobility, self-care, usual activities, pain/discomfort, and anxiety/depression. Additionally, patients completed a visual Analog scale (VAS) ranging from 0 to 100, reflecting their general health status on the consultation day. The data were collected during the nutritional assessment appointment at the Pharmacy Service.

### 2.5. Statistical Analysis

The quantitative variables were analyzed according to standard descriptive statistics, mean, median, range, and standard deviation, while qualitative variables were expressed as frequency and percentage.

The Shapiro–Wilk test was performed to analyze the assumption of normality. For continuous variables that followed a normal distribution, Student’s t-test was used. In cases where normality was not met, the Mann–Whitney test was applied. No adjustments for multiple comparisons were applied, as the statistical analyses were independent, and no multiple post hoc tests were conducted. For categorical variables, the Chi-square test was used, or Fisher’s exact test, when the expected frequencies in the contingency table were less than or equal to 5. A *p*-value of <0.05 was considered statistically significant. The software used for the analysis was IBM SPSS^®^ Statistics version 23.

## 3. Results

A total of 25 patients (60% women) were included in this study, with a median age of 72 (33–87) years, diagnosed with non-small-cell lung cancer (NSCLC): 96% had adenocarcinoma and 4% had squamous cell carcinoma. All patients included in the study were at an advanced stage of the disease (72% stage IV; 28% stage III). The median duration of osimertinib treatment, considering the start date and the date of the nutritional assessment, was 472 (42–1526) days.

The usual dosage of osimertinib was 80 mg every 24 h. Fifty-two percent of the patients in the study had their dosage adjusted due to toxicity: 60 mg every 24 h (40%), 40 mg every 24 h (8%), and 40 mg every 48 h (4%). The observed dose-limiting toxicities (DLT) were diarrhea (20%), mucositis (8%), folliculitis (8%), nausea (4%), thrombocytopenia (4%), paronychia (4%), and elevated transaminases (4%).

After applying the GLIM criteria, malnutrition was diagnosed in 36% of the patients. Regarding the phenotypic criteria, 28% of the patients reported having experienced involuntary weight loss of more than 10% in at least 6 months, 4% had low BMI, 8% met the criteria for low muscle mass, and 4% exhibited more than one phenotypic criterion. Regarding the etiological criteria, 40% of the patients reported a reduction in food intake or assimilation, and 100% of the patients met the criterion of inflammation or acute/chronic disease due to the diagnosis of advanced-stage NSCLC.

Table 1 details the anthropometric parameters and dynamometry results for all study subjects. The classification of patients according to the BMI reference values from the Spanish Society for the Study of Obesity (SEEDO) showed that 48% of the patients had adequate weight, 36% were overweight, and 16% were classified as obese. Twenty percent of the patients showed dynapenia.

Body composition analysis using bioelectrical impedance analysis (BIA) was performed in 23 patients (92%), and the results are detailed in Table 2. BIA could not be performed on one patient due to the presence of a pacemaker, which contraindicates the procedure, and another patient refused to undergo the test due to fear. Eight percent of the patients had reduced muscle mass.

Regarding the diagnosis of sarcopenia, the SARC-F test was applied to 100% of the patients, following the EWGSOP2 algorithm. Sixteen percent of the patients scored four or higher and were classified as at risk of sarcopenia. After measuring handgrip strength using dynamometry, 20% of the patients had dynapenia, 8% were classified as presarcopenic due to their low muscle mass, and 4% were diagnosed with sarcopenia as they presented both characteristics.

The differences observed in the anthropometric parameters and body composition between patients with DLT and those without DLT are detailed in Table 3. Handgrip strength, measured by dynamometry, showed statistically significant differences between the groups (22 kg vs. 34 kg; *p* = 0.015). Patients with DLT had lower values for height, current weight, usual weight, and calf circumference, as well as greater weight loss, although there were no statistically significant differences between the groups.

Regarding body composition, statistically significant differences were observed in FFM (kg) (41.5 vs. 49.2; *p* = 0.039) and the FFMI (kg/m^2^) (16.4 vs. 18.1; *p* = 0.022), with lower values in patients with DLT. FM (kg) was higher in patients with DLT, although the difference was not statistically significant (23.4 vs. 19.6; *p* = 0.318).

**Figure 1:**
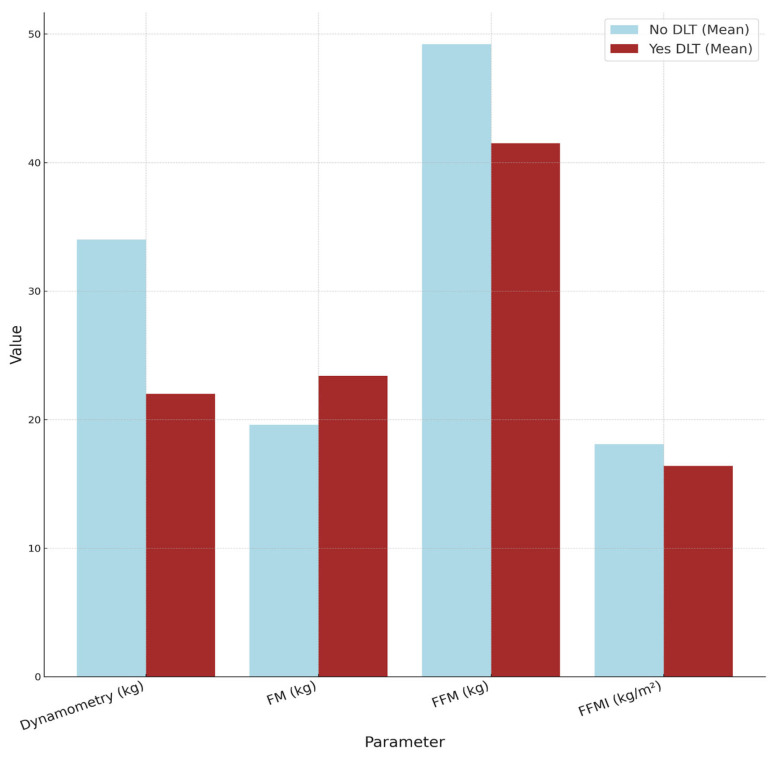
Comparison of body composition and muscle strength parameters between patients with and without DLT. DLT: dose-limiting toxicity; FM: fat mass; FFM: fat-free mass; FFMI: fat-free mass index.

Figure 1 illustrates these differences, showing that patients with DLT presented lower mean values for dynamometry, FFM, and the FFMI compared to those without DLT, while FM was slightly higher in the DLT group.

Of the patients diagnosed with malnutrition, 66.7% experienced DLT, although this association was not statistically significant (*p* = 0.41). Regarding dynapenia, all affected patients experienced DLT, showing a statistically significant correlation (*p* = 0.039). One hundred percent of the patients with presarcopenia or sarcopenia experienced DLT without reaching statistical significance (*p* = 0.48 and *p* = 1.00, respectively).

Quality of life, measured through the visual analog scale (VAS) of the EQ-5D, showed a mean score of 67.7 with a standard deviation of 19.3. In the five dimensions of the EQ-5D, the most frequently reported problems among patients were anxiety/depression (64%), pain/discomfort (60%), and mobility issues (56%). No statistically significant differences in quality of life were observed between patients with and without malnutrition (*p* = 0.886) (Table 4).

## 4. Discussion

In this study, we evaluated the nutritional status, body composition, and prevalence of sarcopenia in patients with NSCLC treated with osimertinib. Although malnutrition in NSCLC patients has been documented in other studies [1,2], this work offers a new perspective, as no studies to date have explored the relationship between the use of osimertinib and the nutritional status of these patients.

The prevalence of malnutrition in our cohort was 36%, which is consistent with published data showing malnutrition in lung cancer patients ranging between 35% and 68% [1,2]. In our study, there was a trend toward a higher risk of DLT in malnourished patients, although this association did not reach statistical significance. This reinforces the idea that malnutrition is a prevalent and significant issue in NSCLC patients and may negatively impact treatment tolerance and effectiveness.

Our analysis of body composition revealed that patients who experienced DLT had significantly lower FFM and FFMI. These results align with previous studies showing a relationship between low muscle mass and increased toxicities during treatment with TKIs, especially sorafenib [4,5,6,7,8,9]. However, no studies to date have specifically analyzed the association between body composition and osimertinib toxicity, highlighting the relevance of our work.

A recent study reported that treatment with osimertinib is associated with significant weight loss in NSCLC patients with EGFR mutations. It was observed that 46% of patients experienced a weight loss of 5% or more within the first 6 to 12 months of treatment, which correlated with a significant decrease in overall survival. These findings emphasize the importance of monitoring weight and body composition in patients receiving osimertinib to prevent and manage early potential adverse effects related to muscle mass loss and malnutrition [14].

Regarding sarcopenia, our study observed a prevalence of 4%, which is notably lower than the 43% reported in a recent meta-analysis of NSCLC patients [15]. This discrepancy may be attributed to differences in sarcopenia diagnostic criteria, specific characteristics of our study population, or methodologies used to assess body composition [15]. Additionally, the prevalence of presarcopenia (8%) and dynapenia (20%) in our cohort is challenging to compare with previous studies due to the limited available data on these conditions in NSCLC patients. Notably, while all patients with sarcopenia and presarcopenia, as well as most patients with dynapenia, experienced DLT, only dynapenia reached statistical significance.

Quality of life, assessed using the EQ-5D visual analog scale (mean score of 67.7), did not differ significantly between malnourished and non-malnourished patients. This unexpected finding may be influenced by confounding factors such as advanced disease stage (72% were stage IV), the presence of specific side effects, such as gastrointestinal adverse events and dermatological issues, and high rates of psychosocial issues (anxiety and depression in 64% of patients). Future studies should address these factors to better understand the impact of malnutrition on quality of life and consider incorporating personalized psychosocial and nutritional interventions.

Our study presents several limitations that should be considered when interpreting the results. First, the sample size was determined based on the total number of patients treated with osimertinib in our healthcare area who met the inclusion and exclusion criteria, resulting in 25 patients. Since all available patients were included, no prior sample size calculation or effect size analysis was performed, which reduced the statistical power to detect significant differences. Second, the cross-sectional design of our study precludes establishing causal relationships between nutritional status and toxicities. Although our results suggest a possible relationship, other factors, such as disease stage, specific symptoms, concomitant treatments, physical activity, systemic inflammation, and overall nutritional status, may have influenced the outcomes. Moreover, the absence of a control group comprising NSCLC patients who are not treated with osimertinib or who receive alternative therapeutic regimens makes it difficult to isolate the treatment effect. Finally, potential information bias in the assessment of weight loss and dietary changes due to reliance on patient interviews rather than comprehensive medical records may have led to inaccuracies.

To address these limitations, we recommend that future studies adopt a longitudinal approach that allows for the temporal monitoring of patients’ nutritional and functional evolution during treatment. This type of study could provide stronger evidence on whether nutritional deterioration precedes the development of toxicities or if there is a bidirectional relationship between these effects. Moreover, increasing the sample size through multicenter studies would improve the statistical power and generalizability of the findings. In addition, future research should include a control group comprising NSCLC patients who are not treated with osimertinib or who receive alternative therapeutic regimens to better isolate the treatment effect. Incorporating objective measures of physical activity, such as activity trackers or validated self-report questionnaires, and using medical records to document weight loss, dietary intake, and concomitant treatments would further enhance the reliability of the data, providing a more complete view of the impact of nutrition on the response to osimertinib treatment.

## 5. Conclusions

This study highlights the prevalence of malnutrition and the importance of body composition in patients with non-small-cell lung cancer treated with osimertinib. Although no statistically significant relationship was found between malnutrition and dose-limiting toxicities, the results suggest a trend that reinforces the need to address nutritional status in this patient group. In particular, the identification of low muscle mass and dynapenia as factors associated with a higher risk of toxicity underscores the importance of evaluating not only weight and body mass index but also muscle functionality as part of the clinical follow-up of NSCLC patients.

These findings have significant implications for clinical practice, as they support the integration of early and personalized nutritional intervention strategies into the multidisciplinary management of NSCLC. Implementing systematic assessments of nutritional status and muscle function could help optimize treatment tolerance, minimize adverse effects, and improve patients’ quality of life. Additionally, by identifying dynapenia as a possible predictor of toxicity, our results suggest that incorporating nutritional support strategies, such as dietary supplementation programs and resistance training, could prevent complications and improve therapeutic outcomes in these patients.

Furthermore, this study paves the way for future research to determine whether targeted nutritional interventions can reduce osimertinib-related toxicities and ultimately improve treatment adherence and survival in this population. The relevance of these findings supports the need for comprehensive care, where oncologists, pharmacists, and nutrition specialists work together to optimize the clinical management of NSCLC.

Nevertheless, due to the cross-sectional nature of this study and the limited sample size, further research with longitudinal designs and larger cohorts is necessary to confirm these observations and establish effective nutritional support strategies for the treatment of NSCLC.

## Figures and Tables

**Table 1:** Anthropometric parameters.

Parameter	Mean	Median	SD	Minimum	Maximum
Height (m)	1.61	1.60	0.09	1.41	1.77
Current Weight (kg)	66.76	68.00	10.09	51.00	85.30
Usual Weight (kg)	70.00	72.00	10.26	52.00	90.00
Weight Loss (%)	4.29	4.52	8.67	−17.08	19.69
BMI (kg/m^2^)	25.78	25.11	3.74	19.97	34.49
Calf Circumference (cm)	35.24	35.00	2.96	30.00	42.00
Dynamometry (kg)	27.76	24.00	11.80	12.00	56.00

BMI: body mass index; SD: standard deviation.

**Table 2:** Body composition parameters.

Parameter	Mean	Median	SD	Minimum	Maximum
FM (kg)	21.6	19.8	8.79	5.90	37.5
FFM (kg)	45.2	45.2	9.17	31.20	64.4
FFMI (kg/m^2^)	17.2	17.5	1.90	12.54	20.8

FM: fat mass; FFM: fat-free mass; FFMI: fat-free mass index; SD: standard deviation.

**Table 3:** Anthropometric and body composition parameters according to the presence of osimertinib DLT.

Parameter	DLT	Mean	Median	SD	*p*-Value
Height (m)	No	1.64	1.67	0.10	0.117
	Yes	1.58	1.58	0.08	
Current Weight (kg)	No	68.57	68.95	9.73	0.400
	Yes	65.08	68.00	10.51	
Usual Weight (kg)	No	71.33	71.50	9.19	0.545
	Yes	68.77	72.00	11.40	
Weight Loss (%)	No	3.76	4.35	7.15	0.775
	Yes	4.78	5.22	10.14	
BMI (kg/m^2^)	No	25.57	24.63	4.02	0.798
	Yes	25.96	25.29	3.61	
Calf Circumference (cm)	No	36.08	36.50	2.15	0.177
	Yes	34.46	34.00	3.45	
Dynamometry (kg)	No	34.00	32.50	12.60	0.015
	Yes	22.00	20.00	7.59	
FM (kg)	No	19.6	18.00	10.16	0.318
	Yes	23.4	20.00	7.30	
FFM (kg)	No	49.2	48.30	9.50	0.039
	Yes	41.5	41.40	7.39	
FFMI (kg/m^2^)	No	18.1	18.00	1.56	0.022
	Yes	16.4	16.10	1.83	

BMI: body mass index; FM: fat mass; FFM: fat-free mass; FFMI: fat-free mass index; DLT: dose-limiting toxicity; SD: standard deviation.

**Table 4:** VAS score of the EQ-5D questionnaire according to malnutrition diagnosis.

Malnutrition	*n*	Mean	Median	SD	*p*-Value
Yes	9	67.8	70.0	17.9	0.886
No	16	67.6	70.0	20.6	

VAS: visual analog scale; *n*: number of patients; SD: standard deviation.

## Data Availability

Data are contained within the article.
